# Galactoligosaccharide and a prebiotic blend improve colonic health and immunity of adult dogs

**DOI:** 10.1371/journal.pone.0238006

**Published:** 2020-08-28

**Authors:** Mariana Fragoso Rentas, Raquel Silveira Pedreira, Mariana Pamplona Perini, Larissa Wünsche Risolia, Rafael Vessecchi Amorim Zafalon, Isabella Corsato Alvarenga, Thiago Henrique Annibale Vendramini, Júlio Cesar Carvalho Balieiro, Cristiana Ferreira Fonseca Pontieri, Marcio Antonio Brunetto

**Affiliations:** 1 Pet Nutrology Research Center, Nutrition and Animal Production Department, School of Veterinary Medicine and Animal Science, University of São Paulo–USP, Pirassununga, Brazil; 2 Nutritional Development Center, Grandfood Indústria e Comércio Ltda (Premier Pet), Dourado, Brazil; 3 Grain Science and Industry Department, Kansas State University, Manhattan, KS, United States of America; University of Kentuky, UNITED STATES

## Abstract

This study aimed to evaluate the effects of two prebiotics in different concentrations on nutrient digestibility, fermentative products and immunological variables in adult dogs. Twenty-four adult dogs were randomly divided into six blocks according to their metabolic body weights (BW^0.75^); within these groups, dogs were randomized to four treatments: control without prebiotics (CO); inclusion of 0.5% prebiotic blend Yes-Golf (B1); inclusion of 1.0% galactooligosaccharide (GOS); and inclusion of 1.0% prebiotic blend Yes-Golf (B2). The experiment lasted 30 days, with 20 days adaptation and 10 days stool and blood collection. Results were analyzed for normality and means were separated by ANOVA and adjusted by the Tukey test at the significance level of 5.0%. Prebiotic supplementation had no effect on apparent digestibility coefficients (ADC), total stool production and fecal scores (p > 0.05). Prebiotics evaluated also did not alter fecal pH, nor the concentrations of ammonia, lactic acid, short chain fatty acids (SCFA) and most fecal branched chain fatty acids (BCFA) (p > 0.05). The addition of GOS decreased the concentration of iso-valeric acid (p = 0.0423). Regarding immunological variables, concentrations of fecal IgA were not influenced by the treatments. Treatments GOS and B2 increased the total number of polymorphonuclear cells, as well as the oxidative burst in relation to treatments B1 and CO (p < 0.0001). Treatment B2 improved the rate of S. aureus phagocytosis in relation to CO (p = 0.0111), and both the GOS and B2 treatments had a better index for E. coli phagocytosis than the CO treatment (p = 0.0067). In conclusion, there was indication that both prebiotics GOS and B2 at 1.0% inclusion improved the immunity of healthy dogs.

## Introduction

Prebiotics represent some of the most common functional ingredients used in pet foods. These can be defined as substrates used selectively by host microorganisms that confer gut health benefits [1]. Prebiotics may be present in dietary ingredients or may be added through concentrated exogenous sources [2, 3]. Their main function is the modulation of native host microbiota [4] by stimulating beneficial bacterial growth and(or) activating their metabolism in the intestinal tract. Bacteria considered beneficial may reduce pathogenic strands through various mechanisms and improve intestinal health [3].

Besides promoting direct positive effects on intestinal health, prebiotics can indirectly improve the animal's immune system by stimulating the growth of lactic acid-producing bacteria. These bacteria produce substances with immunostimulatory properties, which interact with the immune system and stimulate cytokine production, mononuclear cell proliferation, macrophage phagocytosis and induction of synthesis of larger amounts of immunoglobulins [5].

Galactooligosaccharides (GOS) are prebiotics synthesized from lactose transgalactosylation. Recent studies attribute to these oligosaccharides a number of potential health benefits [6]. Galactooligosaccharides stimulate *Bifidobacterium* proliferation in the colon, which suppress the activity of putrefying bacteria by antagonistic effect and reduce the formation of toxic metabolites [7, 8]. Other common prebiotics used in companion animal nutrition include manannoligosaccharides (MOS), fructoligosaccharides (FOS) and beta glucans.

In order to optimize the isolated effects of certain prebiotics it is possible to use these as blends. When incorporated into the animal's diet, blends can modulate the microbiota, improve the animal’s intestinal health and immunity, and confer additional benefits of each different prebiotic [9]. Thus, the present study aimed to determine the effects of GOS and prebiotic blend on nutrient digestibility, fecal fermentation end products and immunological variables of adult dogs.

## Materials and methods

This study was in agreement with the ethical principles in animal experimentation adopted by the Brazilian College of Animal Experimentation (COBEA) and the Ethical Principles in Animal Research established by the Ethic Committee on Animal Use of the School of Veterinary Medicine and Animal Science at the University of São Paulo (CEUA/FMVZ). The study was approved by the CEUA under the protocol number 5359160216.

### Location, facilities and animals

The experiment was conducted at the Premier Pet Nutrition Development Center (CDN Premier Pet; Dourado, SP, Brazil). Twenty-four mixed-breed healthy male and female dogs were selected, with a mean age of 4.0 ± 2.0 years and body condition score between 4 and 5 [10]. Dogs were housed in individual kennels with dimensions of either 2.0 x 5.60m or 2.0 x 4.90m equipped with litter boxes. Fresh water was offered *ad libitum*. During the collection period, dogs remained in the same kennels and were individually released for one hour in exercise areas, accompanied by a student. When dogs defecated, feces were immediately harvested.

### Diets and experimental design

The animals were separated into six blocks according to their metabolic weights (BW^0.75^). Within each block, dogs were randomized to four treatments: CO (control treatment, without prebiotic addition), GOS (control treatment with 1.0% galactooligosaccharides), B1 (control treatment with 0.5% Yes-Golf® prebiotic blend addition), B2 (control treatment with 1.0% Yes-Golf® prebiotic blend; Table 1), following a randomized block design. The commercial blend Yes-Golf® had MOS, FOS, GOS and beta glucan in its composition (Table 1). Both prebiotics GOS and Yes-Golf® are marketed by Yes Sinergy do Brasil Agroindustrial LTDA (Campinas, São Paulo, SP, Brazil). Experimental diets were formulated according to the nutritional recommendations by the *Fédération européenne de líndustriedes aliments pour animaux familiers* [11]. During diet production, the ingredients were weighed, ground, mixed and then extruded at Premier Pet Factory Unit (Dourado, São Paulo, SP, Brazil). All ingredients used in the production of experimental diets were obtained from a single batch to minimize variability. Prebiotics were added to kibbles post-extrusion during the coating process, along with the powder palatant.

**Table 1:** Ingredient composition of experimental foods.

Ingredients (%)	CO^1^	GOS^2^	*B1*^3^	*B2*^4^
Prebiotic	0.0	1.0	0.5	1.0
Poultry viscera meal	36.0	36.0	36.0	36.0
Rice	30.0	30.0	30.0	30.0
Corn	21.41	21.41	21.41	21.41
Poultry fat	8.2	8.2	8.2	8.2
Liquid palatant^5^	2.0	2.0	2.0	2.0
Dry palatant^5^	1.0	0.0	0.5	0.0
Potassium chloride	0.43	0.43	0.43	0.43
Salt	0.3	0.3	0.3	0.3
Mineral and vitamin premix^6^	0.52	0.52	0.52	0.52
Antifungal	0.1	0.1	0.1	0.1
Antioxidant (BHA and BHT)	0.04	0.04	0.04	0.04

^1^CO (control food, without prebiotic addition)

^2^GOS [control food with 1.0% galactooligosaccharides added (min. 380g/kg)]

^3^Blend 0.5% [control food with the addition of 0.5% Yes-Golf® blend: beta glucans (min. 150g/kg), frutooligosaccharides (mín. 120g/kg), galactooligosaccharides (min. 72g/kg), glucomannan (min. 210g/kg), mananoligossacharides (min. 60g/kg)]

^4^Blend 1.0% (control food with the addition of 1.0% Yes-Golf® blend).

^5^Dry matter basis. Pork and chicken liver hydrolysate.

^6^Nutrient addition per kilogram: Iron 100mg, copper 10mg, manganese 10mg, zinc 150mg, iodine 2mg, selenium 0.3mg, vitamin A 18000UI, vitamin D 1200UI, vitamin E 200UI, thiamine 6mg, riboflavin 10mg, pantothenic acid 40mg, niacin 60mg, pyridoxine 6mg, folic acid 0.30mg, vitamin B12 0.1mg and choline 2000mg.

The feeding study lasted for a total of 30 days, with the first 20 days of adaptation to foods. From day 21 to 25, total fecal samples were collected for measurement of apparent digestibility. From day 26 to 29, fresh feces were collected into 2mL vials and immediately frozen at -80ºC for later determination of fermentation products. On the last day (day 30), 5mL blood was collected from jugular venipuncture according to [12] recommendations, and stored in polystyrene boxes with ice until immunological variables were measured on the same day. The animals were fed twice a day to maintain their metabolic energy requirement, which was based on the [13] energy requirement prediction equation [95x (BW)^0.75^ = kcal per day]. The amount of daily food consumption by dogs was calculated by subtracting any remaining kibbles from the food offered. The animals were weighed weekly and food offered adjusted when necessary, in order to maintain body weight (BW) and body condition score (BCS).

### Apparent digestibility of nutrients and fecal score

Dietary ADCs were determined by the total fecal collection method according to [12]. In summary, food consumption was recorded daily and total feces were collected for five days. Stools were weighed immediately after collection, placed in individual plastic bags, and stored in a freezer (-15°C) for further analysis. At the end of the collection period, feces were thawed and homogenized, composing a single sample per animal (fecal pool). These were then weighed and dried in a forced ventilation oven (320-SE, FANEM, São Paulo, Brazil) at 55ºC for at least 72 hours, until moisture content decreased below 10%. The pre-dried stools and diets were then ground in a knife mill (MOD 340, ART LAB, São Paulo, SP, Brazil) with a 1mm sieve, and stored in plastic jars at ambient temperature until laboratory analyses.

The dry matter (DM), crude protein (CP), acid-hydrolyzed fat (AHF), ash and crude fiber (CF) contents were determined in both feces and food (Table 2) according to the methodologies described by [12]. Nitrogen-free extracts (NFE) were calculated using the formula: NFE = 100 - (%CP + %EEHA + %CF + %ash). All proximate analyses were performed at the Multiuser Laboratory of Animal Nutrition and Bromatology of the Department of Nutrition and Animal Production of FMVZ/USP (University of São Paulo, Pirassununga, SP, Brazil). The ADCs of DM, OM, CP, EEHA, CF and NNE were calculated according to the equation below:

$$ADC=\frac{Nutrient\;intake-nutrient\;output}{nutrient\;intake}$$

**Table 2:** Chemical composition of experimental foods.

Item (%)	Treatments			
	CO^1^	GOS^2^	B1^3^	B2^4^
Dry matter	91.95	91.80	91.76	91.76
	**Chemical composition on a DMB**^**5**^			
Ash	6.47	6.61	6.72	6.85
Crude protein	27.46	26.93	25.28	27.22
Acid hydrolyzed fat	14.17	14.53	14.32	14.37
Crude fiber	6.30	6.40	5.97	5.83
Nitrogen-free extract	45.6	45.53	47.71	45.73

^1^CO (control food, without prebiotic addition)

^2^GOS [control food with 1.0% galactooligosaccharides added (min. 380g/kg)]

^3^Blend 0.5% [control food with the addition of 0.5% Yes-Golf® blend: beta glucans (min. 150g/kg), frutooligosaccharides (min. 120g/kg), galactooligosaccharides (min. 72g/kg), glucomannan (min. 210g/kg), mananoligossacharides (min. 60g/kg)]

^4^Blend 1.0% (control food with the addition of 1.0% Yes-Golf® blend).

^5^Dry matter basis.

All stools collected during the digestibility study were scored for consistency on a 5-point scale (0 to 5), with formed and firm (ideal) stools being between 3 and 4 [14].

### Fermentation products

Fecal pH was determined by homogenizing one gram of fresh feces with 9mL distilled water, and introducing the electrode into 2 points of the solution [15] using a digital benchtop pH meter (Digimed, DM-20, Quimis do Brasil Ltda; São Paulo, SP, Brazil).

Fecal ammonia nitrogen and short chain fatty acids (SCFA) were determined on fresh homogenized fecal samples, collected within 15 minutes after defecation. Immediately upon collection, three grams of duplicated fecal samples for each parameter measured (fecal ammonia and SCFA) were mixed with 9mL 16% formic acid. The mixture was kept in a refrigerator for seven days and stirred daily. Subsequently, the mixture was centrifuged at 5,000 rpm for 15 minutes at 15°C three times, discarding the pellet. The supernatants were extracted, identified, and stored at -15°C. For fecal ammonia nitrogen quantification, the extracts were thawed at room temperature, 2mL aliquots were diluted in 13mL distilled water, and then processed in a nitrogen distiller according to [16]. These analyzes were performed at the Multiuser Laboratory of Animal Nutrition and Bromatology of the Department of Nutrition and Animal Production of FMVZ/USP (University of São Paulo; Pirassununga, SP, Brazil).

For SCFA determination, the last supernatant was transferred to an Eppendorf tube (Eppendorf Flex-Tubes Microcentrifuge Tubes, Sigma-Aldrich, Darmstadt, Germany) before freezing and storage. At the end of all periods, all samples were thawed and centrifuged at 14,000 rpm (Rotanta 460 Robotic, Hettich, Tuttlingen, Germany) for 15 min. Fecal SCFA concentrations were analyzed by gas chromatography (SHIMADZU, model GC–2014, Kyoto, Japan), according to [17] and adapted by [18]. The analysis was performed using a 30m × 0.53mm glass column (Stabilwax ®, Restek, Bellefonte, EUA) at 145ºC, and nitrogen as carrier gas at a flow rate of 8.01mL/min. The working temperatures were: injection, 250°C; column, 145°C (at a speed of 20°C/min); and flame ionization detector, 250°C. These analyzes were performed at the FZEA / USP Ruminal Fermentability Laboratory (University of São Paulo; Pirassununga, SP, Brazil).

Lactic acid was measured according to the methodology described by [19]. Briefly, three grams of fresh feces (collected within 30 minutes of defecation) were homogenized and mixed with 6mL distilled water (1:2 w/v). These were read at 565nm (500 to 570nm) using a spectrophotometer (QUICK-Lab, DRAKE Eletrônica Comércio LTDA, São José do Rio Preto-SP, Brazil), and compared against a 0.08% lactic acid standard. The lactic acid analysis was performed at the Multiuser Laboratory of Animal Nutrition and Bromatology of the Department of Nutrition and Animal Production of FMVZ/USP (University of São Paulo; Pirassununga, SP, Brazil).

### Immunological variables

Immunological assays included blood leukocyte, total polymorphonuclear cells, intracellular production of reactive oxygen species (ROS) and blood leukocyte phagocytosis. These analyzes were performed by flow cytometry (FACS Calibur TM-Becton Dickinson Immunocytometry System TM cytometer; San Diego, CA, USA), according to the methodology described by [20]. On the last day of the experiment, approximately 3mL of blood was collected from each dog through jugular venipuncture and placed in a tube containing heparin (BD Vacutainer® lithium heparin, BD, New Jersey, USA). The determination of ROS and phagocytosis test required a pre-treatment of samples. For the measurement of ROS, 100μL of blood were mixed with 200μL of 2.7 dichlorodihydrofluoresceinacetate (DCFH-DA; 0.3mM) in a polypropylene tube, and then incubated at 37°C for 30 minutes.

Phagocytosis tests were conducted by adding 2μL of Staphylococcus aureus or Escherichia coli already labeled with fluorescent reagent Alexa Fluor® conjugate to the polypropylene tubes. These were incubated for 60 minutes at 37ºC, and then the reactions were stopped by the addition of 2,000μL cold ethylenediaminetetraacetic acid (EDTA) solution (3mM). The tubes were centrifuged at 250 x g for eight minutes and the supernatants discarded. Then, samples were homogenized, and the red cells were hypotonically lysed with saline solution (first at 0.2% dilution, to lyse red cells and then at 1.6% to stop cell lysis). After this procedure, samples were centrifuged twice. Finally, the samples were read on a FACS Calibur TM flow cytometer (Becton, Dickinson and Company; San Diego, CA, USA) connected to an Apple Macintosh computer (Apple factory in Fremont; California, USA) with the CELLQUEST® - Becton Dickinson Immunocytometry System TM program (San Diego, CA, USA). A total of 10,000 cells were acquired from each tube and the data obtained from the readings were analyzed on FlowJo Treestar—vX.0.7 version for Windows software (Treestar; Ashland, OR, USA). These analyzes were performed at the Immunodiagnostic Laboratory of the Department of Veterinary Clinic of FMVZ/USP (São Paulo, SP, Brazil).

In addition to the immunoassays performed on blood, fecal IgA was also determined. For this, 3g of fresh fecal sample (within 30 minutes of defecation) were collected and frozen at -15ºC. Samples were thawed on the day of the analysis and IgA was extracted with saline solution according to [21]. In summary, one gram of feces was weighed, added to 10mL extraction buffer [0.01M PBS; pH 7.4; 0.5% Tween 80 (Sigma-Aldrich, Poole, Dorset, UK) and 0.05% sodium azide], and homogenized with a vortex mixer (Vortex basic 220, Kasvi, São José dos Pinhais, PR, Brazil). The suspension was centrifuged at 1,500 x g for 20 minutes at 5°C, and two milliliters of the supernatant were transferred to a 5mL conical tube containing 20μL protease inhibitor cocktail (Sigma-Aldrich; Darmstadt, Germany). The solution was again homogenized and centrifuged at 15,000 x g for 15 minutes at 5°C, and the supernatant transferred to an Eppendorf tube (Eppendorf Flex-Tubes Microcentrifuge Tubes, Sigma-Aldrich, Darmstadt, Germany) and stored at -20°C. Immunoglobulin A quantitation was performed using a canine IgA ELISA kit (Bethyl Laboratories, Montgomery, TX, U.S.A.) according to the manufacturer's recommendations. The reading was performed on an ELISA Microplate Reader (MRX TC Plus Microplate Reader, Dynex Technologies, Chantilly, VA, U.S.A.) through a 450nm filter at the Laboratory Specialized in Scientific Analysis (LEAC; São Paulo, SP, Brazil).

### Statistical analyses

The results were analyzed using the Statistical Analysis System (SAS Institute Inc. v. 9.1.1, SAS Inst., Cary, NC, 2004) computer program [22]. The normality of the residuals was verified by the Shapiro-Wilk test using the univariate procedure from SAS and the homogeneity of the variances by the F-test. Data which did not meet the statistical assumptions suffered logarithmic transformation or square root. Observations were considered outliers when its studentized residual was above +3.4 or below -3.4. Fecal scores per dog collected for five days were averaged and were subjected to the same parametric test as the other variables. The fixed effect was the diets, and dog blocked by metabolic body weight was the random effect. Finally, analysis of variance was performed by the MIXED procedure from SAS with Tukey adjustment at 5% significance level according to the following statistical model:

Yij=μ+ti+bj+eij

In which:

Yij=dependentvariable;μ=overallmean;ti=fixedeffectoftreatment;bj=fixedblockeffect;eij=residualerror.

## Results

### Apparent digestibility coefficient of nutrients and fecal score

All animals had adequate food intake and no food rejection or diarrhea were reported. There were no outliers, so all observations were kept for statistical analysis. During the experiment, dog weights and body condition scores were monitored and maintained. There was no difference between the treatments in the ADC variables of DM, OM, CP, AHF and NFE (P> 0.05), as well as in wet fecal production and fecal scores (P > 0.05; Table 3).

**Table 3:** Apparent nutrient digestibility coefficients, production and fecal score of dogs (n = 6) fed experimental diets.

Item	Treatments				SEM	*P*
	CO^1^	GOS^2^	B1^3^	B2^4^		
	**Apparent digestibility coefficients (%)**					
Dry matter	83.7	82.0	82.5	82.5	1.12	0.734
Organic matter	87.0	85.6	86.0	86.2	0.93	0.754
Crude protein	86.0	83.8	84.5	84.2	1.38	0.443
Crude fiber	73.5	73.2	73.1	74.0	2.20	0.985
Acid hydrolyzed fat	97.8	97.4	97.9	98.0	0.16	0.102
Nitrogen-free extract	83.4	81.3	82.0	82.4	1.32	0.738
	**Fecal production**					
Fecal score	3.55	3.71	3.55	3.51	0.09	0.273
Fecal production (g/day)	548.8	564.9	533.9	671.5	143.75	0.682

^1^CO (control food, without prebiotic addition)

^2^GOS [control food with 1.0% galactooligosaccharides added (min. 380g/kg)]

^3^Blend 0.5% [control food with the addition of 0.5% Yes-Golf® blend: beta glucans (min. 150g/kg), frutooligosaccharides (min. 120g/kg), galactooligosaccharides (min. 72g/kg), glucomannan (min. 210g/kg), mananoligossacharides (min. 60g/kg)]

^4^Blend 1.0% (control food with the addition of 1.0% Yes-Golf® blend).

### Fermentation products

There was no difference (P > 0.05) in the concentration of most fermentation products measured in the study (fecal pH, lactic acid, ammonia and SCFA; Table 4).

**Table 4:** Fecal pH, lactic acid, ammonia, short chain and branched fatty acids measured in feces of dogs (n = 6) fed the experimental diets.

Item	Treatments				SEM	*P*
	CO^1^	GOS^2^	B1^3^	B2^4^		
Fecal pH	6.75	6.77	6.65	6.58	0.125	0.615
Lactic acid, mmol/Kg of DM^5^	9.16	14.39	13.57	14.24	1.642	0.116
Ammonia, mmol/Kg of DM	115.47	129.95	153.00	136.73	15.733	0.325
	**Short chain fatty acids, mmol/Kg DM**					
Acetic acid	221.01	242.98	268.02	268.02	26.922	0.639
Propionic acid	169.50	172.91	154.99	187.29	18.305	0.572
Butyric acid	48.19	44.45	56.53	53.94	6.555	0.565
Total SCFA^6^	475.38	460.34	432.53	509.26	48.014	0.722
	**Branched chain fatty acids, mmol/Kg DM**					
Valeric acid	2.32	1.49	1.80	1.25	0.651	0.683
Isovaleric acid	10.97	9.78	13.23	10.21	1.098	0.091
Isobutyric acid	8.74	9.72	9.93	9.93	1.134	0.842
Total BCFA^7^	22.04	20.99	24.97	21.40	2.298	0.508

^1^CO (control food, without prebiotic addition)

^2^GOS [control food with 1.0% galactooligosaccharides added (min. 380g/kg)]

^3^Blend 0.5% [control food with the addition of 0.5% Yes-Golf® blend: beta glucans (min. 150g/kg), frutooligosaccharides (mín. 120g/kg), galactooligosaccharides (min. 72g/kg), glucomannan (min. 210g/kg), mananoligossacharides (min. 60g/kg)]

^4^Blend 1.0% (control food with the addition of 1.0% Yes-Golf® blend).

^5^DM, dry matter

^6^SCFA, short chain fatty acids

^7^BCFA, branched chain fatty acids.

### Immunological variables

Total leukocyte percentage, unstimulated ROS production, unstimulated fluorescence intensity (P > 0.05) and fecal IgA were not different among treatments (P > 0.05; Table 5).

**Table 5:** Results of phagocytosis and oxidative burst tests in dogs (n = 6) fed experimental diets.

Item	Treatments				SEM	P
	CO^1^	GOS^2^	B1^3^	B2^4^		
Total leukocytes (%)	78.90	74.70	75.90	82.35	3.347	0.286
Total polymorphonuclear cells (%)	35.38^b^	60.01^a^	41.10^b^	60.26^a^	2.754	<0.0001
ROS basal production^5^ (%)	94.01	97.21	98.93	94.30	1.686	0.248
Basal fluorescence intensity	1740	1294	1281	1451	225.0	0.138
*S*. *aureus* phagocytosis (%)	56.66^b^	73.35^ab^	67.31^ab^	81.10^a^	5.601	0.011
*S*. *aureus* fluorescence intensity	73.21^b^	495.83^a^	152.67^b^	517.33^a^	38.760	<0.0001
*E*. *coli* phagocytosis (%)	46.31^b^	62.88^a^	52.31^ab^	67.15^a^	4.091	0.006
*E*. *coli* fluorescence intensity	151.3^b^	526.2^a^	255.2^b^	510.8^a^	43.627	<0.0001
Fecal IgA	18.55	13.85	10.15	8.74	3.862	0.305

^1^CO (control food, without prebiotic addition)

^2^GOS [control food with 1.0% galactooligosaccharides added (min. 380g/kg)]

^3^Blend 0.5% [control food with the addition of 0.5% Yes-Golf® blend: beta glucans (min. 150g/kg), frutooligosaccharides (min. 120g/kg), galactooligosaccharides (min. 72g/kg), glucomannan (min. 210g/kg), mananoligossacharides (min. 60g/kg)]

^4^Blend 1.0% (control food with the addition of 1.0% Yes-Golf® blend).

^5^ROS, reactive oxygen species

^a.b^Means on the lines followed by equal superscripts do not differ from each other by Tukey's test (p˃0.05).

The percentage of polymorphonuclear cells in relation to total leukocytes was higher in animals that consumed the GOS and B2 diets (P < 0.0001). Regarding the phagocytosis test with gram positive bacteria (*S*. *aureus*), the percentage of cells that phagocytized at least one bacterium was higher in dogs fed the B2 diet than those fed the negative control (CO; P = 0.0111); however, group B2 did not differ from groups GOS and B1 (P > 0.05). The GOS and B2 groups presented higher means than CO in the phagocytosis test with gram negative bacteria (P = 0.0067), and B1 was similar to the extremes. Finally, the oxidative burst, for both gram-positive and gram-negative stimuli GOS and B2 presented higher fluorescence intensity. This translates to a higher amount of ROS produced in dogs fed the GOS and B2 treatments due to more intensive phagocytoses in comparison to those fed the B1 and CO diets. The oxidative burst is measured by flow cytometry through the immunofluorescence intensity emitted by the contact of ROS with the fluorescence reagent of pre-treated bacteria (E. coli or S. aureus). When the bacteria is phagocyted, the cell generates ROS and the color intensity changes. The ROS produced during phagocytosis has antimicrobial activity due to microbial DNA and protein damage [23, 24].

## Discussion

In the present study, the inclusion of prebiotics did not affect ADCs of nutrients, although most studies with prebiotics have observed changes in at least one. For example, [9] observed a decrease in DM, CP and NFE digestibility in dogs supplemented with 1g of MOS per kg of daily body weight for 10 days. Our animals received an average of 0.63g prebiotic per day in treatment B1 and 1.23g in GOS and B2 treatments, which was smaller than the concentration used by [9]. The higher prebiotic inclusion in the work by [9] probably was more effective in increasing the microbial material in the feces, which may have underestimated apparent digestibility of some nutrients. Conversely, the low concentration of prebiotics offered in the present study might not have been enough to produce this effect. This finding was also observed in other studies using a smaller concentration of prebiotics. For example, supplementing dog food with 5g/kg or 1g/kg prebiotics did not change nutrient total tract ADC [25, 26]. Therefore, lower concentrations of prebiotics may not be enough to promote an increase microbial mass and interfere with apparent digestibility. Supplementing dog food with 5g/kg or 1g/kg prebiotics did not change nutrient total tract ADC [25].

The prebiotics tested at their respective concentrations did not alter fecal pH, fecal lactic acid, fecal score, fecal mass and fecal SCFA. Fecal mass and fecal scores results corroborate the findings of [26] and [27], who did not report changes in fecal variables with the use of prebiotics FOS, MOS and yeast cell wall (YCW). Conversely, [28] found a linear increase in fecal mass production of dogs fed increasing levels of YCW (at 0.0, 1.5, 3.0 and 4.5g/kg). The source and concentration of prebiotics, as well as the nutritional matrix of the diet, are important factors that may impact fecal variables [29].

Fecal score can be related to colonic SCFA, which also did not present differences in fecal concentration among treatments. Short-chain fatty acids modulate water absorption by altering the osmotic pressure in the colon, and this effect may be dose-dependent [13]. As a result of excess volatile fatty acids in the colon, loose stools may occur. The maintenance of quality stools is often observed by dog owners and is an important factor on pet food purchasing decisions. Even though the fecal scoring system is a subjective method, it is an important tool for assessing stool quality, especially in prebiotic research [13]. In a study with dogs supplemented with a prebiotic blend (MOS + FOS) [26], did not observe a decrease in pH or an increase in SCFA production [30]. Evaluating different inclusion levels of YCW in dogs also did not find any difference in fermentation products. These results were justified by the rapid absorption of SCFA in the colon.

Prebiotics addition to the food have demonstrated positive stimulation on the immune system. In general, the direct influence of prebiotics occurs by preventing the adhesion of pathogens to the mucus, decreasing the attachment and invasion of these pathogens to the intestinal mucosa. Prebiotics may also stimulate dendritic cells in the gut by increasing cell membrane permeability [31]. These cells are present in the cellular junctions of the intestinal epithelium and help reduce the penetration of pathogens through the epithelial barrier [31].

Indirectly, prebiotics serve as substrate for the intestinal microbiota, shifting its composition towards beneficial bacteria. Saccharolytic bacteria produce SCFA that serve as energy to the intestinal epithelial cell, among other important physiological functions [32]. Short-chain fatty acids may suppress macrophage activity and stimulate Treg cell production [32]. Some intestinal bacterial groups improve systemic immunity by increasing Treg cells both locally and in distant organs such as the spleen and lungs [32]. Besides that, some bacterial groups considered to be potentially pathogenic, such as salmonella and shigella, are unable to ferment prebiotics due to the lack of glycosidic hydrolases and saccharolytic enzymes, reducing their chance of survival. In addition, saccharolytic bacteria and epithelial cells produce antimicrobial peptides that inhibit growth, adhesion and may even lead to apoptosis of pathogenic bacterial groups [31].

Although no effects of feeding prebiotics at the tested concentrations were observed regarding the modulation of fermentation products, there were some positive changes in immunological variables. Dogs fed with 10g/kg of GOS or Yes-Golf® blend (B2) had an increase in the relative number of polymorphonuclear cells, as well as an improvement in the phagocytosis index and an increase in ROS production for both gram-positive and gram-negative bacteria stimuli. Few experiments have assessed the effect of prebiotics on the immune response of healthy dogs. The pioneering study on this field found a higher concentration of lymphocytes in dogs fed the prebiotic treatments (composed of MOS and FOS individually and blended together) compared to the control [26]. In the present experiment the animals fed with treatments GOS and B2 presented higher concentration of polymorphonuclear cells, which are also cells involved in the immunological function against pathogens. A more recent study observed an improvement in phagocytic activity of neutrophils in dogs that consumed 15g MOS per kg of food, which was a concentration much higher than what used in the present study (mean 1.23g/day prebiotic) [33]. To our knowledge, there was only one past experiment which assessed oxidative burst and phagocytosis index in dogs fed prebiotics. In that study, the authors observed an increase in phagocytosis index of dogs supplemented with a yeast cell wall-based prebiotic [30]. Similarly, the immunological activity of dogs supplemented with GOS and B2 improved in the present study.

It has been reported that GOS may improve immune responses through SCFA production [34, 35]. Galactooligosaccharide fermentation by beneficial intestinal microbiota may result in the production of SCFA that can bind to immune component receptors (GPR43) and subsequently affect innate immunity components and inflammatory cells [32]. Supplementation of different prebiotics including GOS to common carp (*Cyprinus carpio*) increased innate immunity, total Ig and significant level of lysozyme [36]. In addition, the effect of prebiotics on immune response has been attributed to the increase in the number of beneficial bacteria in the gut microbial community, such as lactic acid-producing bacteria that have lipopolysaccharide cell walls that can stimulate the immune system [37–39].

In addition to GOS, the blend used in this study contained MOS, FOS and β -glucan which have also shown evidence of immunological improvement. Some specific prebiotic compounds (mannan and glucans) perform antibacterial, antimutagenic, antitumor and antioxidant functions [40]. Besides that, these polysaccharides can stimulate the production and activity of macrophages and neutrophils, which enhance immunity and increase resistance to gram-negative pathogenic bacteria [40–42]. It is proposed that the mannose present on the surface of these polysaccharides may stimulate the production of a mannose-binding lectin, which is fundamental in the phagocytosis process of innate immune responses to microorganisms [43].

The FOS present in treatment B2 may also have influenced the immune system of dogs. It was reported that this prebiotic interacts with Toll-like receptor-2 (TLR2), a membrane surface receptor expressed in macrophages, polymorphonuclear cells (polymorphonuclear leukocytes or granulocytes) and dendritic cells, which results in the activation of immune cells through pathways of signal transduction in humans [44].

Finally, β-glucan can act as an immunostimulant and activate immune cells by binding to their specific lectin-1 receptor. This is a type C lectin receptor expressed on the surface of macrophages [45, 46]. The lectin-1 receptor bound to β-glucan activates macrophages synergistically with TLR2 and its signaling pathway, which induce pro-inflammatory response and TNF-α secretion [47]. However, when β-glucan is administered orally, it may suffer acid hydrolysis in the stomach and lose its immunostimulatory effect [48]. Therefore, the mechanisms related to β-glucan as immunostimulant need to be further studied [35].

According to the results obtained in this experiment, dogs fed the treatments GOS and B2 had an increase in the concentration of polymorphonuclear cells, the number of cells that performed at least one phagocytosis, and in the oxidative burst measured by flow cytometry. Besides that, treatments containing prebiotics did not affect nutrient digestibility and fecal characteristics. In conclusion, the prebiotic blend and GOS at 1% led to the greatest improvements in immunity, suggesting that these prebiotics should be employed at a concentration greater than 1% to promote health.
